# A Novel Voltametric Measurements of Beta Blocker Drug Propranolol on Glassy Carbon Electrode Modified with Carbon Black Nanoparticles

**DOI:** 10.3390/ma14247582

**Published:** 2021-12-09

**Authors:** Izabela Bargiel, Joanna Smajdor, Anna Górska, Beata Paczosa-Bator, Robert Piech

**Affiliations:** Department of Analytical Chemistry and Biochemistry, Faculty of Materials Science and Ceramics, AGH University of Science and Technology, Al. Mickiewicza, 30-059 Kraków, Poland; izabela.bargiel1991@gmail.com (I.B.); smajdorj@agh.edu.pl (J.S.); gorska.anna7@gmail.com (A.G.); paczosa@agh.edu.pl (B.P.-B.)

**Keywords:** propranolol, carbon black, Nafion, voltammetry, modified electrode

## Abstract

A new voltametric method for highly sensitive propranolol (PROP) determination was developed. A glassy carbon electrode modified with a hybrid material made of carbon black (CB) and Nafion was used as the working electrode. The preconcentration potential and time were optimized (550 mV and 15 s), as well as the supporting electrolyte (0.1 mol L^−1^ H_2_SO_4_). For 15 s preconcentration time, linearity was achieved in the range 0.5–3.5 μmol L^−1^ and for 120 s in 0.02–0.14 μmol L^−1^. Based on the conducted calibration (120 s preconcentration time) limit of detection (LOD) was calculated and was equal to 7 nmol L^−1^. To verify the usefulness of the developed method, propranolol determination was carried out in real samples (tablets and freeze-dried urine). Recoveries were calculated and were in the range 92–102%, suggesting that the method might be considered as accurate. The repeatability of the signal expressed as relative standard deviation (RSD) was equal to 1.5% (*n* = 9, PROP concentration 2.5 µmol L^−1^). The obtained results proved that the developed method for propranolol determination might be successfully applied in routine laboratory practice.

## 1. Introduction

Propranolol (PROP) belongs to the group of non-selective β-blockers. Its mechanism of action bases on the inhibition of β1 and β2 receptors. Pharmacological inhibition of these receptors inhibits its stimulation. It limits the influence of epinephrine and norepinephrine on tissues that possess β-receptors (e.g., in the heart, vessels, and bronchi). In practice, β-blockers reduce heart rate and contraction force and lead to reduction of blood pressure. Propranolol might be characterized by a wide range of clinical applications, e.g., treatment of hypertension, primary and secondary prevention of myocardial infarction, prevention of migraine, reduction of anxiety, control of arrhythmias [1,2,3].

From the chemical point of view, propranolol is an organic compound described as 1-[(1-methylethyl) amino]-3-(1-naphthalenyloxy). In the literature various analytical propranolol determination methods have been reported, among them were spectrophotometry [4,5], spectrofluorimetry [6,7], high performance liquid chromatography (HPLC) [8,9,10], and capillary electrophoresis [11,12]. Another method that is commonly used for propranolol determination is voltammetry. In comparison with the above mentioned methods, voltammetry might be characterized by very low detection limit, high sensitivity, low interferences impact, relatively low cost of analysis and no need to use toxic chemicals. The most important part of each voltammetric system is the working electrode (WE). For propranolol determination, different types of solid electrodes were used, e.g., glassy carbon electrode (GCE) [13,14], graphite electrode (GE) [15,16], carbon paste electrode (CPE) [17,18], boron doped diamond electrode (BDDE) [19], and screen printed electrode (SPE) [20,21]. A recent trend in electrochemical methods has focused on the modification of solid electrodes (GCE, CPE, GE, and SPE) in order to improve their performance. Surface modifiers should exhibit certain properties, like, for example, good electrical conductivity, high specific surface area, and easy electron transfer. Therefore, different types of materials might be used for this purpose: carbon nanomaterials [22,23], metal nanoparticles [24,25], conducting polymers [26,27], etc.

However, use of the functionalized carbon nanomaterials such as carbon nanotubes or graphene for voltammetric measurements is associated with the risk of obtaining heterogeneous layers with different nanotubes orientations, which may cause the problem of low repeatability of obtained signals. Carbon nanotubes per se may also differ from each other considering the differences in its activation process or various numbers of active centers or function groups on its surface, that also can affect working conditions. Nowadays, electrode modifiers consisted of noble metals nanoparticles getting more attention, but its manufacturing process is also quite demanding, requiring the usage of strong acid under the conditions that can generate toxic products. Other disadvantage of such solution is the quite high price of such modifiers.

The aim of this work was developing of a new, highly sensitive and simple method for propranolol determination. For this purpose, a glassy carbon electrode modified with carbon black and Nafion was used. Developed modifier is an example of hybrid material (combination of carbon nanomaterial and polymer) that combines advantages of both components. Undoubted advantage of carbon black combined with Nafion as a modifier layer is obtaining wide working surface due to its physical parameters. The consequence of enlarging the electrode surface is clearly visible when comparing the detection limits of voltammetric sensors based on other modification materials. The use of Nafion as a dispersion component results not only in expanding the working surface, but also assures shorter electrode preparation time of about 15 min for the measurement process due to its quick drying process. This significantly improves the measurement process compared to other popular solvents used in electrode preparation, which have to be left to dry completely for a few hours. The simplicity of the proposed sensor and low cost of manufacturing are also crucial factors of choosing such a design solution.

## 2. Experimental

### 2.1. Measuring Apparatus

For all voltametric measurements, a multipurpose Electrochemical Analyzer M161 and the electrode stand M164 (MTM-ANKO, Krakow, Poland) with the EAGRAPH software (1.0, Krakow, Poland) were used. The standard three-electrode voltammetric quartz cell with volume of 20 mL was composed of a glassy carbon electrode modified with carbon black as the working electrode (CBGC), a double junction silver chloride reference electrode Ag/AgCl/KCl (3 mol L^−1^), and a platinum rod as an auxiliary electrode. Homogenization of the supporting electrolyte was ensured using magnetic Teflon-coated bar (stirring speed of about 500 rpm). pH-meter (N-512 elpo, Polymetron, Wroclaw, Poland) was used to measure solutions pH value. All experiments were carried out at room temperature.

### 2.2. Chemicals

Standard stock solution of propranolol (Sigma Aldrich, Darmstadt, Germany) was obtained by dissolving an appropriate weight of standard in proportion of water and ethanol (1:1) and stored in fridge (10 mL, 0.01 mol L^−1^). Sulfuric acid (96%) was purchased from Merck (Darmstadt, Germany), methanol (99%) and ethanol (96%) was purchased from POCH (Gliwice, Poland). The Triton X-100 was purchased from Windsor Laboratories Ltd. (Kingston, Jamaica). Interferents: citric acid, lactose monohydrate, starch, magnesium stearate, talc, cellulose, titanium dioxide, glucose, caffeine, ascorbic acid, uric acid, acetaminophen were purchased from Merck (Darmstadt, Germany). Carbon black nanoparticles with the surface area of 100 m^2^ g^−1^ and average particle size of 30 nm were obtained from 3D-nano (Kraków, Poland). The ion-exchange polymer Nafion (5% solution in a mixture of lower aliphatic alcohols) was obtained from Sigma-Aldrich (Darmstadt, Germany). Freeze-dried human urine was purchased from Medichem (Hobokem, NJ, USA). All reagents were of analytical grade and used without further purification. All solutions were prepared with double-distilled water.

### 2.3. Pharmaceutical Sample Preparation

Pharmaceutical samples such as propranolol WZF (Polfa Warszawa, Warszawa, Poland) and propranolol Accord (Accord Healthcare, London, UK) were investigated to measure the propranolol content. Pharmaceuticals were obtained from a local pharmacy. For measurements, samples were prepared by crushing three tablets in a mortar and quantitatively transferring to the volumetric flask (10 mL) and dissolving in water and ethanol (1:1). After complete dissolving and homogenization solution was ready for analysis. Solutions with lower concentrations were prepared daily.

The amount of propranolol in the samples was measured by the standard addition method and validated with the recovery parameter. 

### 2.4. Urine Sample Preparation

Urine sample was prepared by dissolution of freeze-dried human urine with 5 mL of double distilled water and shaking on the ultrasonic washer (Emag, Leipzig, Germany) until complete dissolution of the powder. Then 900 µL of urine and 100 µL of methanol were transferred to the Eppendorf flask (1.5 mL) and stirred on the table centrifuge (Eppendorf, Hamburg, Germany) at 2000 rpm for 1 min. Measurements were performed in the supporting electrolyte consisting of 0.1 mol L^−1^ H_2_SO_4_ with the addition of 100 µL of previously prepared urine sample using the standard addition method and validated with recovery parameter. 

### 2.5. Standard Procedure of Measurements

The differential pulse voltammetry (DPV) technique was applied for highly sensitive quantitative measurements of propranolol. The electrode was coated with 10 µL of homogenized carbon black solution layer daily (carbon black suspended in Nafion, 1 mg mL^−1^). After preparation, glassy carbon electrode modified with carbon black nanoparticles and Nafion was used for propranolol (PROP) determination in the supporting electrolyte consisting of 0.1 mol L^−1^ H_2_SO_4_ (pH 1.8, total volume of 10 mL). Voltammograms were registered in the potential range from 500 mV to 1275 mV, with preconcentration potential E_acc_ of 550 mV (preconcentration time t_acc_ = 15 s). Other instrumental parameters of DPV technique are as follow: potential step E_s_ = 4 mV, pulse amplitude dE = 50 mV, time step potential 20 s (10 ms waiting time t_w_ and 10 ms sampling time t_s_). 

## 3. Results and Discussion

### 3.1. Voltammetric Characterization of Glassy Carbon Electrode Modified with Carbon Black 

The parameters of carbon black surface on the glassy carbon electrode were investigated in 1 mmol L^−1^ potassium ferricyanide (Fe(CN)_6_^−3^/Fe(CN)_6_^−4^, solution in 1 mol L^−1^ KCl (both from POCH, Gliwice, Poland) using cyclic voltammetry. The range of the scan rate values was from 10 to 250 mV s^−1^. The glassy carbon electrode modified with carbon black nanoparticles in Nafion working surface where the PROP oxidation process takes place was calculated using the dependence between the ferricyanide peak current and the square root of the scan rate. In order to compare the performance of the electrodes, this parameter was calculated both for modified and unmodified electrode. For the electrode modified by Nafion and carbon black, the size of active surface was of about 0.1058 cm^2^, whereas for the unmodified electrode surface the size was significantly lower of about 0.0152 cm^2^, which indicates that the size of modified electrode working surface is approximately seven times higher than unmodified. 

**Figure 1:**
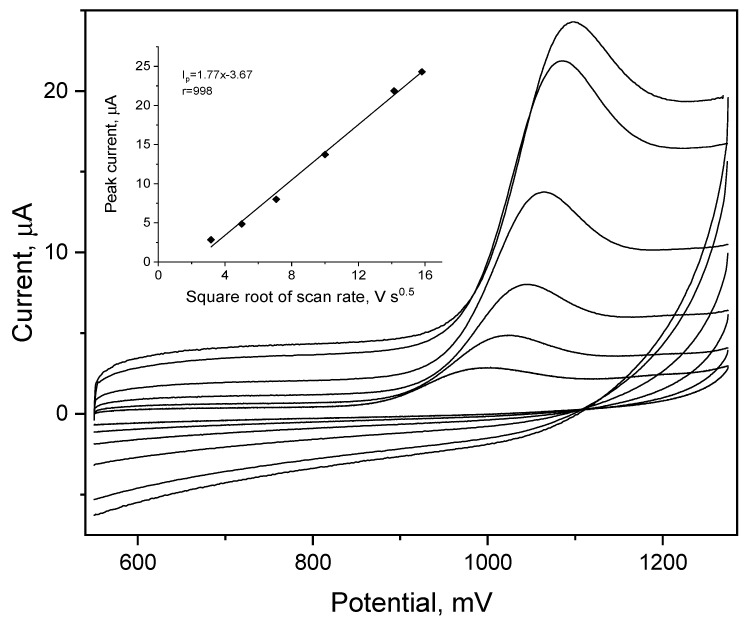
Cyclic voltammograms of 10 µmol L^−1^ propranolol in 0.1 mol L^−1^ H_2_SO_4_ (pH 1.8) measured on the glassy carbon electrode modified with carbon black nanoparticles. Scan rate values: 10, 25, 50, 100, 200, and 250 mV s^−1^.

**Scheme 1:**
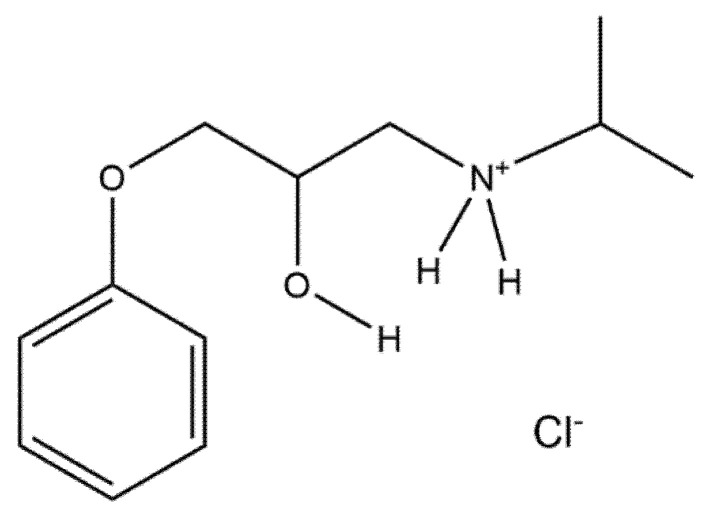
Cationic form of propranolol formed during the electrochemical reaction.

The propranolol behavior was investigated on the CBGC electrode using a cyclic voltammetry technique. Measurements were performed in the supporting electrolyte with addition of 10 µmol L^−1^ PROP. The effect of the scan rate changing in the range from 10 to 250 mV s^−1^ on propranolol oxidation process is presented in Figure 1. The absence of the reduction peak in the cathodic scan implies that propranolol oxidation process on CBGC electrode is irreversible. In order to explain the mechanism of PROP oxidation, the dependences of its peak current versus the scan rate and the square root of the scan rate were plotted. The linear correlation was obtained from the peak potential on the square root of the scan rate plot, that suggests that the propranolol oxidation process takes place by diffusion. The propranolol oxidation process on the glassy carbon electrode modified by carbon black and Nafion is connected with the reaction on the Nafion layer. Considering the propranolol pKa value of 9.42 and the supporting electrolyte with 0.1 M H_2_SO_4_, the reaction group of propranolol exists in cationic form (Scheme 1). The positively charged PROP exchanges protons with the sulphonic group of Nafion, which improves its efficiency of accumulation on the electrode surface. 

The propranolol preconcentration on the glassy carbon electrode modified by the carbon black takes part in the way of adsorption, but the transport from the Nafion modifier layer to the electrode surface is a diffusion-controlled process. The electrode surface modified by carbon black nanoparticles due to its physical properties is characterized by bigger active surface, which allows to accumulate more analyte than on the glassy carbon electrode surface.

Moreover, the plot of the peak current vs. logarithm of the scan rate was developed, with obtained linear regression equation of:

(1) $$E_{k}=0.0133\ln v+0.0267\;\left[V\right],\;r=0.999$$

Considering the obtained values of the regression equation and assuming that the oxidation process of propranolol is irreversible, it is possible to calculate the number of electrons exchanged during the electrode reaction using the Laviron equation [28]:

(2) $$E_{k}=E^{0}+\left(\frac{RT}{\alpha nF}\right)\ln\left(\frac{RTk^{0}}{\alpha nF}\right)+(\frac{RT}{\alpha }nF)\ln v$$

where *α* is the transport coefficient, *k*^0^ is the electrochemical rate constant, *n* is the number of exchanged electrons, *v* is the scan rate value, *E*^0^ is the formal potential, *T* is temperature value, *F* is the Faraday constant and *R* is the gas constant. Assuming *α* value as 0.5 and the value of the α*n* coefficient equal to 0.97, the number of the electrons that participate in the electrochemical propranolol oxidation could be calculated as 2.

The number of electrons exchanged during the propranolol oxidation reaction can also be determinate using following equation:

(3) αn=0.048|Ep−Ep12|

The *αn* value calculated from the equation was equal to 0.96. Assuming *α* as 0.5, the number of electrons exchanged during the oxidation reaction could be calculated as 2, which confirms previous calculations result. 

**Figure 2:**
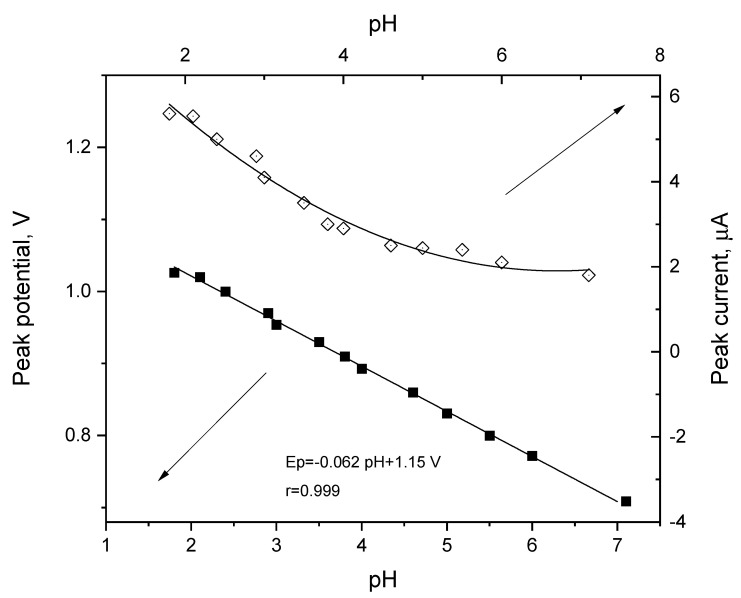
Propranolol peak and potential dependence on supporting electrolyte pH.

To clarify the oxidation mechanism, investigation of the amount of proton that participates in the oxidation process was performed using different pH values of the supporting electrolyte in the range from 1.8 to 7.1 (Figure 2). The propranolol peak was shifting toward more positive potentials along with decreasing pH values. The dependence of the peak potential value versus the supporting electrolyte pH is linear, according to the following equation.

(4) $$E_{p}=0.062\;\mathrm{pH}+1.15\;V$$

**Scheme 2:**
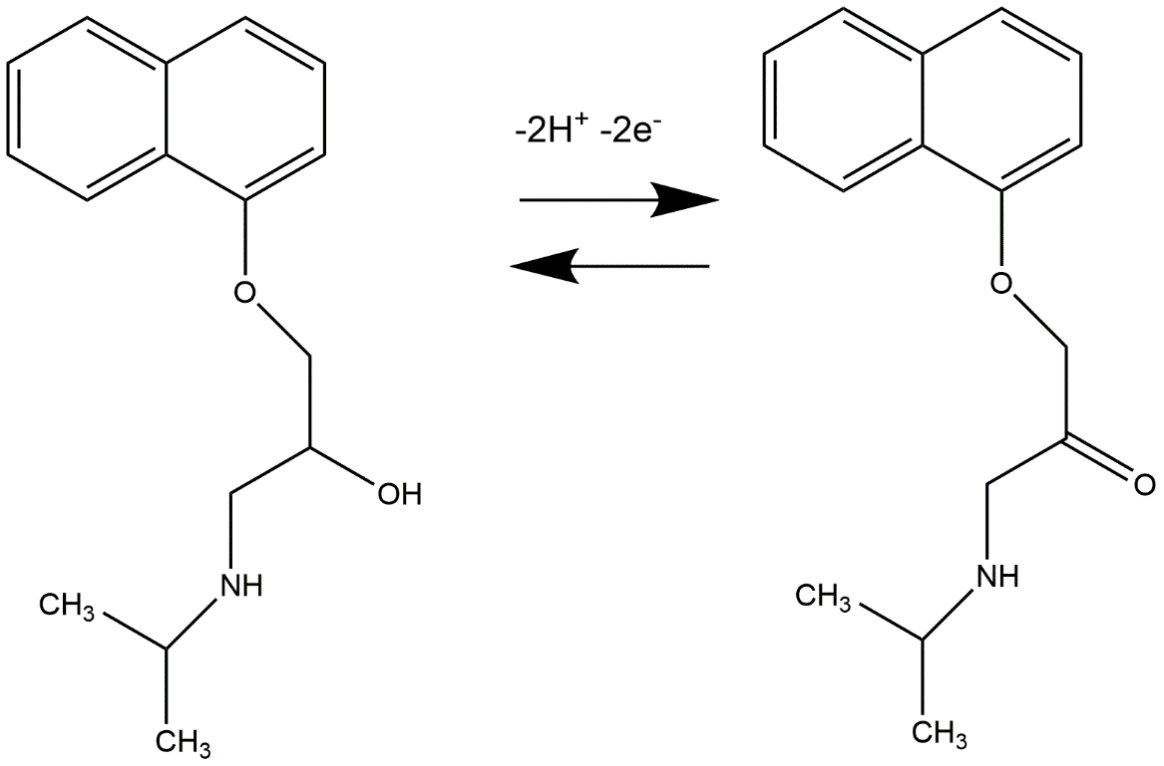
Possible propranolol oxidation mechanism on glassy carbon electrode modified with carbon black nanoparticles.

The value of the slope equals to 0.062 V pH^−1^, which implies that the amount of exchanged electrons and protons is equal during the propranolol oxidation. The proposed mechanism of possible propranolol oxidation on CBGC electrode is presented on Scheme 2. 

A comparison in Linear Sweep Voltammetry (LSV) propranolol measurements between modified and unmodified glassy carbon electrode was performed (Figure not included). A linear correlation between propranolol peak current and square root of scan rate was observed, which indicates that its oxidation process on bare glassy carbon electrode is also diffusion controlled. The plot of the peak current vs logarithm of the scan rate was developed, with the obtained linear regression equation.

Considering the obtained values of the regression equation and assuming that the oxidation process of propranolol is irreversible, it was possible to calculate the number of electrons exchanged during the electrode reaction. Assuming *α* value as 0.5 and the value of the *αn* coefficient equal to 0.64, the number of the electrons that participates in the electrochemical propranolol oxidation could be calculated as 1.

### 3.2. Influence of Modifier Layer Volume on Propranolol Peak 

**Figure 3:**
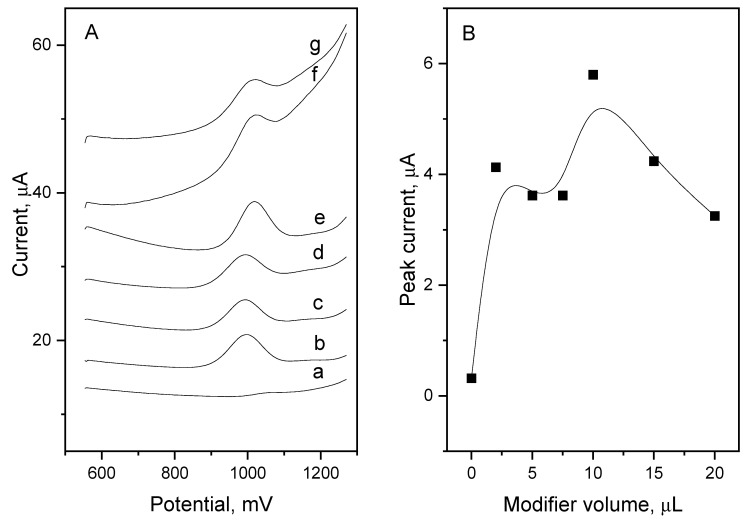
Influence of modifier layer volume on propranolol peak; (**A**) voltammograms obtained for modifier amount of (a) 0 µL, (b) 2 µL, (c) 5 µL, (d) 7.5 µL, (e) 10 µL, (f) 15 µL, and (g) 20 µL and (**B**) the value of corresponding peak current.

In order to examine the influence of modifier volume applied on the glassy carbon electrode surface on the propranolol signal, the appropriate experiment was conducted. The ion-exchange properties of the Nafion depends strongly on the film thickness. Too thick film may decrease the diffusion of the propranolol to the electrode surface, where the exact electrochemical reaction occurs, therefore the obtained signal decreases in its size. Thus, optimizing the amount of modifier layer on the electrode surface is necessary. For this purpose, each GC electrode was modified with a different volume of CB-Nafion dispersion: 0, 2, 5, 7.5, 10, 15, 20 µL (Figure 3). As it might be observed, modification of GCE significantly improved propranolol signal. During the measurements, a few parameters were considered, such as the capacitive current value, the relation of the peak current to the background current, and also the peak shape and its good distinction from the background current. Considering all these parameters, the most favorable characteristic of the propranolol peak with the highest peak current (5.78 µA) was obtained for the layer of 10 µL, therefore this amount of carbon black modification layer was chosen as optimal for further studies. In comparison, peak current register on bare GCE was equal to 0.29 µA, which means that by modification, propranolol signal was improved almost 20 times.

### 3.3. Influence of Preconcentration Time and Potential on Propranolol Peak 

To provide high sensitivity of the performed measurements, the influence of preconcentration potential and time on the propranolol peak values was investigated. The preconcentration potential was investigated in the range from −100 to 750 mV (data not included). Examined values did not significantly affect the potential and current of propranolol peak, therefore the 550 mV was chosen as a propranolol preconcentration potential in furthers studies. 

**Figure 4:**
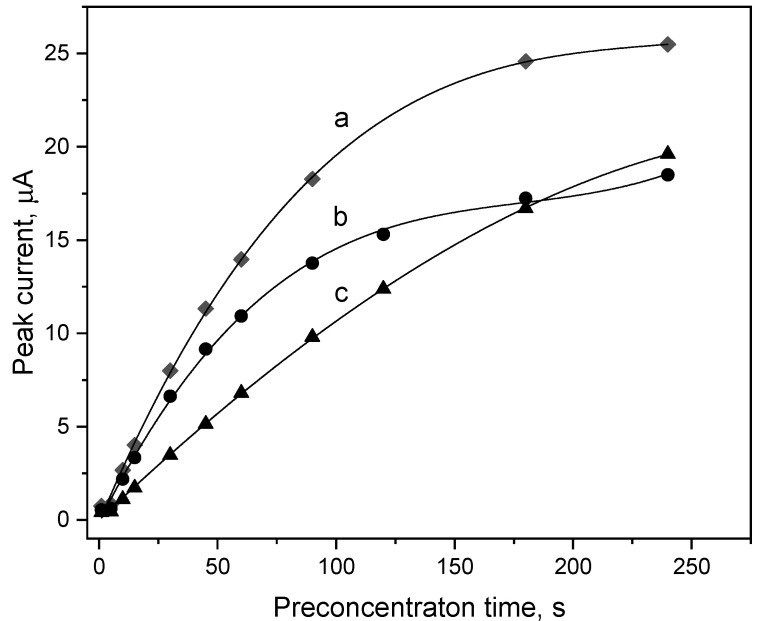
Dependence of the propranolol peak current on preconcentration time in the range from 0 to 240 s for (a) 10 µmol L^−1^ (b) 5.4 µmol L^−1^, and (c) 2 µmol L^−1^ propranolol concentration in 0.1 mol L^−1^ H_2_SO_4_ (pH 1.8).

The plot of relationship between propranolol peak current value and preconcentration time value obtained on CBGC electrode is presented in Figure 4. For all investigated PROP concentration values, increasing the preconcentration time results in an increased value of peak current. The maximum obtained peak current was for the propranolol concentration of 10 μmol L^−1^ and it was equal to 25.49 μA (t_acc_ 240 s), while for 2.0 μmol L^−1^ it was equal to 19.69 (t_acc_ 240 s). For the analytical performance studies time of 15 s was picked to accumulate the analyte on the CBGC electrode surface, in order to ensure the good quality of the signal obtained simultaneously with a short duration of a single analysis.

### 3.4. Influence of Supporting Electrolyte on Propranolol Peak

To maintain optimal conditions of propranolol determination, its peak properties (concentration of 10 µmol L^−1^) were examined in miscellaneous base electrolytes, such as: 0.1 mol L^−1^ ammonia buffer (pH 8.2, Chempur, Piekary Śląskie, Poland), 0.1 mol L^−1^ acetate buffer (pH 3.8, Chempur, Piekary Śląskie, Poland), 0.1 mol L^−1^ H_2_SO_4_ (pH 1.8), 0.1 mol L^−1^ KH_2_PO_4_ (Merck, Darmstadt, Germany), 0.1 mol L^−1^ KCl, and 0.1 mol L^−1^ HCl (Figure not included). Signals obtained in a supporting electrolyte consisted of sulfuric acid characterized by the optimal properties of obtaining the propranolol peak, considering the relationship of its peak current value and background current value. The signal obtained in this environment was also characterized by good peak shape and high repeatability. Furthermore, to ensure the best possible measurement conditions, the influence of sulfuric acid concentration in a range of 0.025 to 0.5 mol L^−1^ on the propranolol peak was examined (Figure not included). The change in this parameter was shown to not significantly affect the maximum current, therefore the supporting electrolyte of 0.1 mol L^−1^ H_2_SO_4_ was selected for later studies. 

### 3.5. Influence of Potential Interferents on Propranolol Peak

Study of interferences is an important part of developing a new analytical method. It allows to determine the influence of potential interferents (that might be found in sample’s matrix) on analyte signal. In the experiment, the influence of the following metals on the propranolol signal was investigated: Mg(II), Ca(II), Na(I), K(I) (50 μmol L^−1^ added), Cu(II), Pb(II), Cd(II), Zn(II), Mo(IV), Mn(II) (5 μmol L^−1^ added). Moreover, organic compounds and potential ingredients of the pharmaceutical formulation and urine were tested, such as: citric acid (50 μmol L^−1^ added), lactose monohydrate, starch, magnesium stearate, talc, cellulose, titanium dioxide, glucose, caffeine, ascorbic acid, uric acid, acetaminophen (20 μmol L^−1^ added), and Triton X-100 (2.5 ppm added). Each measurement was carried out in 0.1 mol L^−1^ H_2_SO_4_, the preconcentration potential and time were equal to 550 mV and 15 s, respectively, the propranolol concentration was 10 μmol L^−1^. Among the tested substances only a few of them had influence on propranolol signal. In the case of Cd(II) and Mo(II), their concentration equal to 5 μmol L^−1^ caused a 15% and 13% decrease in the peak current, respectively. The presence of Mg(II) resulted in a 25% increase in the signal (concentration 50 μmol L^−1^). Cu(II) ions caused a 22% decrease in the peak current when its concentration was equal to 5 μmol L^−1^. Remaining interferents had no or negligibly small influence on propranolol signal. In Table 1 changes in propranolol peak current before and after interferents dosing are presented.

### 3.6. Calibration and Real Samples Studies

**Figure 5:**
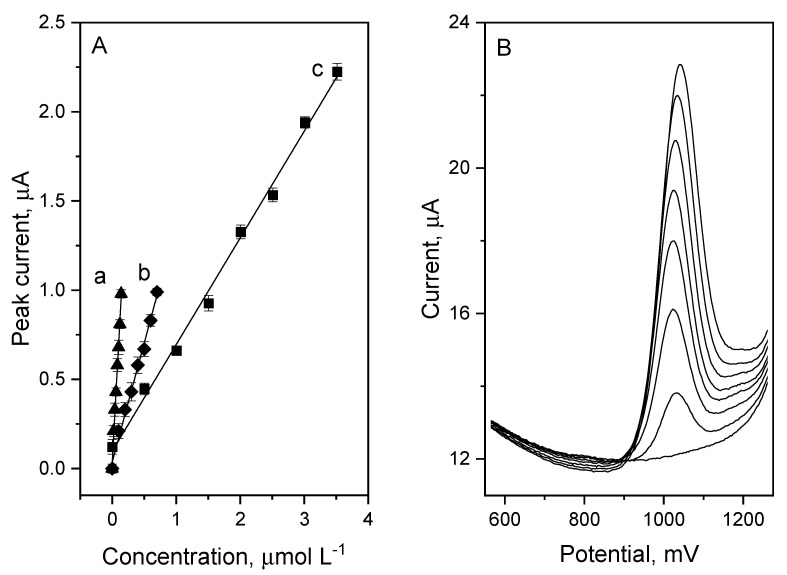
DPV calibration curves of propranolol registered for preconcentration times (a) 2 min, (b) 1 min, and (c) 15 s in 0.1 mol L^−1^ H_2_SO_4_ (pH 1.8) (**A**) and corresponding voltammograms for preconcentration time of 1 min and concentration range 0 to 0.7 µmol L^−1^ (**B**).

Propranolol DP voltammograms of 0.02 to 3.5 μmol L^−1^ with a preconcentration time in the range from 15 to 120 s was registered and presented in Figure 5. 

The linear dependence between PROP concentration and peak current value for short preconcentration time of 15 s was in the range from 0.5 up to 3.5 μmol L^−1^, with the detection limit of 0.12 × 10^−6^ mol L^−1^ (signal to noise relation = 3) and sensitivity of 0.59 μA μM^−1^ (Ip = 0.598x + 0.098, R = 0.998). In order to achieve lower detection limit, parameter of preconcentration time was elongated to 120 s. Obtained calibration curve with linearity from 0.02 to 0.14 μmol L^−1^ let to accomplish the detection limit of 0.007 × 10^−6^ mol L^−1^ (signal to noise relation = 3) and sensitivity of 6.58 μA μM^−1^ (Ip = 6.583x + 0.042, R = 0.997). The reproducibility of the presented propranolol determination method was calculated from the obtained voltammograms and specified as RSD with the value of 1.5% for 9 repetition of measurements of 2.5 µmol L^−1^ propranolol concentration. Comparison of propranolol detection limits for its different determination methods reported in the literature is showed in Table 2.

To assess its validity, the proposed method was applied to the sensitive propranolol determination in authentic pharmaceutical samples containing the studied drug and freeze-dried human urine sample using the standard addition method. Two commonly accessible drugs: Propranolol WZF (10 mg of propranolol per tablet) and Propranolol Accord (10 mg of propranolol per tablet) were investigated. The sample preparation for analysis was as described in point 2.3. The results obtained with the recovery parameter are presented in Table 1. The value of recovery parameter ranged between 92 and 102% suggests the usefulness of the proposed method for highly sensitive propranolol determination in pharmaceutical samples. 

**Figure 6:**
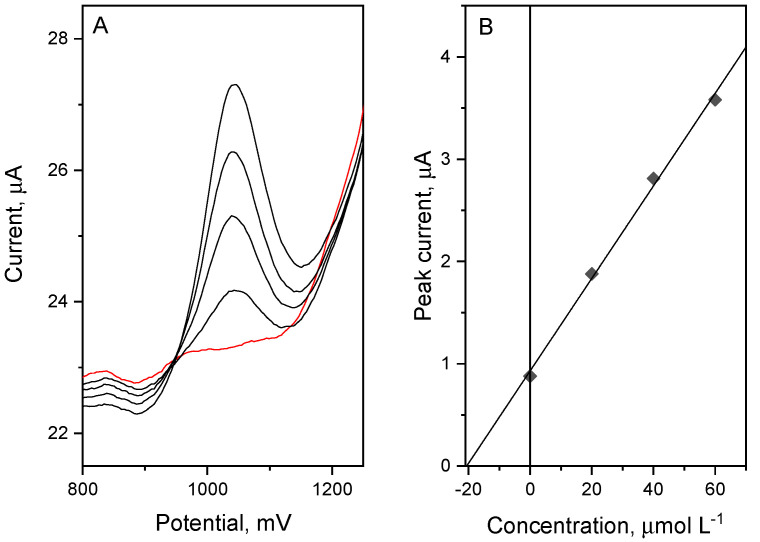
Voltammograms of propranolol determination in urine sample (urine curve marked as red) (**A**) with corresponding calibration curve (**B**).

In addition, a human freeze-dried urine sample was tested in order to check the suitability of the method for sensitive determination of propranolol in human body fluids. The urine sample was prepared as described in point 2.4 and the determination of propranolol was performed using the standard addition method. The results obtained with the recovery parameter measured for each medication are presented in Table 3. The value of recovery parameter ranged between 97 and 106%, and suggests the usefulness of proposed method for high sensitive propranolol determination in urine samples. The sample of the obtained voltammograms with corresponding calibration plot is presented in Figure 6. The PROP peak obtained in urine using glassy carbon electrode modified by carbon black was well shaped and clearly distinguished from the background. By expanding the preconcentration time value, it is possible to reach the propranolol concentration values that are noticed in the real urine sample collected from the patients (1 μg mL^−1^). For the preconcentration time of 45 s, obtained detection limit was of about 18.2 µmol L^−1^.

## 4. Conclusions

In this work, voltametric method of highly sensitive propranolol determination is presented. For the first time of propranolol determination, glassy carbon electrode modified with hybrid nanomaterial based on carbon black and Nafion was used as working electrode. In comparison to previously reported electrode modifiers for highly sensitive propranolol determination, developed sensor might be characterized by ease of its preparation, low-cost, and excellent analytical performance. The conditions for the determination of propranolol were optimized: supporting electrolyte 0.1 mol L^−1^ H_2_SO_4_, preconcentration potential, and time equal to 550 mV and 15 s, respectively. Based on conducted calibrations, LOD values were calculated and were equal to 120 nmol L^−1^ for 15 s preconcentration time and 7 nmol L^−1^ for 120 s preconcentration time. Signal repeatability calculated as RSD was equal to 1.5% (*n* = 9, propranolol concentration 2.5 µmol L^−1^). To verify the usefulness of the developed method, the propranolol concentration was measured in two commercially available pharmaceutical products and in freeze-dried human urine sample. The obtained recovery parameter was in the range 92–106%, which suggests that the method might be assumed to be accurate. Considering the presented results, it might be concluded that the developed voltametric method for propranolol determination using carbon black/Nafion modifier layer could be a useful tool in routine laboratory practice.

## Figures and Tables

**Table 1:** Propranolol peak current change in presence of interferents.

Interferent\Concentration of Interferent	Peak Current Value, µA					Signal Change, %
	0 µmol L^−1^	5 µmol L^−1^	20 µmol L^−1^	50 µmol L^−1^	2.5 ppm	
Mg (II)	5.59	-	-	6.99	-	+25
Ca (II)	5.50	-	-	5.87	-	+7
Na (I)	5.52	-	-	5.74	-	+4
K (I)	5.56	-	-	5.90	-	+6
Cu (II)	5.57	4.35	-	-	-	−22
Pb (II)	5.55	5.77	-	-	-	+4
Cd (II)	5.59	4.75	-	-	-	−15
Zn (II)	5.63	5.35	-	-	-	−6
Mo (IV)	5.49	4.77	-	-	-	−13
Mn (II)	5.09	5.01	-	-	-	−2
Citric acid	5.58	-	-	5.40	-	−3
Lactose monohydrate	5.56	-	5.40	-	-	−3
Starch	5.49	-	5.43	-	-	−1
Magnesium stearate	5.56	-	5.56	-	-	0
Talc	5.58	-	5.47	-	-	−2
Cellulose	5.54	-	5.43	-	-	−2
Titanium dioxide	5.62	-	5.63	-	-	0
Glucose	5.63	5.29	-	-	-	−6
Caffeine	5.61	-	5.22	-	-	−7
Ascorbic acid	5.60	-	5.43	-	-	−3
Uric acid	5.57	-	5.46	-	-	−2
Acetaminophen	5.58	-	5.36	-	-	−4
Triton X-100	5.79	-	-	-	5.18	−11

**Table 2:** Comparison of other propranolol determination methods.

Method	Detection Limit	Source
Spectrophotometry	0.34 µmol L^−1^	[5]
RP-HPLC ^1^	1.04 µmol L^−1^	[8]
SWV (GC/MWCNTs) ^2^	26 nmol L^−1^	[13]
SWV (AgNP-IL-FG-NF/GCE) ^3^	17 nmol L^−1^	[14]
SWV (EPPG/Graphen/CP) ^4^	20 nmol L^−1^	[15]
CV (TiO_2_/MWCNT/PGE) ^5^	21 nmol L^−1^	[16]
SWV (BDDE) ^6^	0.18 µmol L^−1^	[19]
DPV (SPE) ^7^	13 nmol L^−1^	[20]
SWV (C:N electrode) ^8^	0.75 µmol L^−1^	[29]
Polarography	5 nmol L^−1^	[30]
Spectrofluorimetry	11.9 nmol L^−1^	[31]
Spectrofluorimetry	30.8 nmol L^−1^	[32]
LC/MS ^9^	0.19 nmol L^−1^	[33]
Chemiluminescence	3.4 µmol L^−1^	[34]
Chemiluminescence	0.14 µmol L^−1^	[35]
SWV (MWCNT/SR) ^10^	78 nmol L^−1^	[36]
CV (CPE/CuO) ^11^	2.91 µmol L^−1^	[37]
DPV (GC/CB)	7 nmol L^−1^	This work

^1^ Reversed-phase chromatography; ^2^ square wave voltammetry with glassy carbo electrode modified with multiwalled carbon nanotubes; ^3^ square wave voltammetry with glassy carbon electrode modified with functionalized-graphene, ionic liquid and silver nanoparticles; ^4^ square wave voltammetry with edge plane pyrolytic graphite electrode modified with graphene and conductive polymer; ^5^ cyclic voltammetry with pencil graphite electrode modified with TiO_2_ and multiwalled carbon nanotubes; ^6^ square wave voltammetry with boron doped diamond electrode; ^7^ differential pulse voltammetry with screen printed electrode; ^8^ square wave voltammetry with nitrogen-containing tetrahedral amorphous carbon; ^9^ liquid chromatography coupled with mass spectrometry; ^10^ square wave voltammetry with multiwalled carbon nanotubes, graphite and silicone rubber electrode; ^11^ cyclic voltammetry with copper-oxide nanoparticle modified carbon paste electrode.

**Table 3:** Results of propranolol determination in pharmaceutical samples and human urine.

Sample	PROP Added, mg/Tablet	PROP Found ± mg/Tablet	Recovery, %
Propranolol WZF	0	11.5 ± 0.2	-
	15	13.8 ± 0.3	92
	30	30.9 ± 0.2	100
	45	44.9 ± 0.2	100
Propranolol Accord	0	11.7 ± 1.2	-
	15	15.4 ± 0.3	102
	30	28.9 ± 0.5	96
	45	45.6 ± 0.3	101
**Sample**	**PROP added, µg/mL**	**PROP found** **± µg/mL**	**Recovery, %**
Urine diluted 100×	0	Not detected	-
	6	6.36 ± 0.9	106
	12	11.7 ± 0.8	97
	18	18.5 ± 0.5	103
	24	23.8 ± 0.4	99

## Data Availability

The data presented in this study are available on request from the corresponding author.

## References

[1] Bylund D.B. (2015). Propranolol. Reference Module in Biomedical Sciences.

[2] El-Shabrawi M., Hassanin F. (2011). Propranolol Safety Profile in Children. Curr. Drug Saf..

[3] Al-Majed A.A., Bakheit A.H.H., Abdel Aziz H.A., Alajmi F.M., AlRabiah H. (2017). Propranolol. Profiles Drug Subst. Excip. Relat. Methodol..

[4] El-Ries M.A., Abou Attia F.M., Ibrahim S.A. (2000). AAS and spectrophotometric determination of propranolol HCl and metoprolol tartrate. J. Pharm. Biomed. Anal..

[5] El-Emam A.A., Belal F.F., Moustafa M.A., El-Ashry S.M., El-Sherbiny D.T., Hansen S.H. (2003). Spectrophotometric determination of propranolol in formulations via oxidative coupling with 3-methylbenzothiazoline-2-one hydrazone. Farmaco.

[6] El-Abasawi N.M., Attia K.A.M., Abo-serie A.A.M., Morshedy S., Abdel-Fattah A. (2018). Simultaneous determination of rosuvastatin and propranolol in their binary mixture by synchronous spectrofluorimetry. Spectrochim. Acta Part A Mol. Biomol. Spectrosc..

[7] Madrakian T., Afkhami A., Mohammadnejad M. (2009). Simultaneous spectrofluorimetric determination of levodopa and propranolol in urine using feed-forward neural networks assisted by principal component analysis. Talanta.

[8] Imam S.S., Ahad A., Aqil M., Sultana Y., Ali A. (2013). A validated RP-HPLC method for simultaneous determination of propranolol and valsartan in bulk drug and gel formulation. J. Pharm. Bioallied Sci..

[9] Salman S.A.B., Sulaiman S.A., Ismail Z., Gan S.H. (2010). Quantitative determination of propranolol by ultraviolet HPLC in human plasma. Toxicol. Mech. Methods.

[10] Kim H.K., Hong J.H., Park M.S., Kang J.S., Lee M.H. (2001). Determination of propranolol concentration in small volume of rat plasma by HPLC with fluorometric detection. Biomed. Chromatogr..

[11] Micke G.A., Costa A.C.O., Heller M., Barcellos M., Piovezan M., Caon T., de Oliveira M.A.L. (2009). Development of a fast capillary electrophoresis method for the determination of propranolol-Total analysis time reduction strategies. J. Chromatogr. A.

[12] Zhou X., Li X., Zeng Z. (2006). Solid-phase microextraction coupled with capillary electrophoresis for the determination of propranolol enantiomers in urine using a sol-gel derived calix[4]arene fiber. J. Chromatogr. A.

[13] Oliveira G.G., Azzi D.C., Vicentini F.C., Sartori E.R., Fatibello-Filho O. (2013). Voltammetric determination of verapamil and propranolol using a glassy carbon electrode modified with functionalized multiwalled carbon nanotubes within a poly (allylamine hydrochloride) film. J. Electroanal. Chem..

[14] Santos A.M., Wong A., Fatibello-Filho O. (2018). Simultaneous determination of salbutamol and propranolol in biological fluid samples using an electrochemical sensor based on functionalized-graphene, ionic liquid and silver nanoparticles. J. Electroanal. Chem..

[15] Gupta P., Yadav S.K., Agrawal B., Goyal R.N. (2014). A novel graphene and conductive polymer modified pyrolytic graphite sensor for determination of propranolol in biological fluids. Sens. Actuators B Chem..

[16] Dehnavi A., Soleymanpour A. (2021). Titanium Dioxide/Multi-walled Carbon Nanotubes Composite Modified Pencil Graphite Sensor for Sensitive Voltammetric Determination of Propranolol in Real Samples. Electroanalysis.

[17] Broli N., Vasjari M., Vallja L., Duka S., Shehu A., Cenolli S. (2021). Electrochemical determination of atenolol and propranolol using a carbon paste sensor modified with natural ilmenite. Open Chem..

[18] Gaichore R.R., Srivastava A.K. (2014). Electrocatalytic determination of propranolol hydrochloride at carbon paste electrode based on multiwalled carbon-nanotubes and γ-cyclodextrin. J. Incl. Phenom. Macrocycl. Chem..

[19] Sartori E.R., Medeiros R.A., Rocha-Filho R.C., Fatibello-Filho O. (2010). Square-wave voltammetric determination of propranolol and atenolol in pharmaceuticals using a boron-doped diamond electrode. Talanta.

[20] Khairy M., Khorshed A.A. (2020). Simultaneous voltammetric determination of two binary mixtures containing propranolol in pharmaceutical tablets and urine samples. Microchem. J..

[21] Khorshed A.A., Khairy M., Banks C.E. (2019). Electrochemical determination of antihypertensive drugs by employing costless and portable unmodified screen-printed electrodes. Talanta.

[22] Yin H., Ma Q., Zhou Y., Ai S., Zhu L. (2010). Electrochemical behavior and voltammetric determination of 4-aminophenol based on graphene-chitosan composite film modified glassy carbon electrode. Electrochim. Acta.

[23] Xu Q., Wang S.-F. (2005). Electrocatalytic Oxidation and Direct Determination of L-Tyrosine by Square Wave Voltammetry at Multi-Wall Carbon Nanotubes Modified Glassy Carbon Electrodes. Microchim. Acta.

[24] Maringa A., Mugadza T., Antunes E., Nyokong T. (2013). Characterization and electrocatalytic behaviour of glassy carbon electrode modified with nickel nanoparticles towards amitrole detection. J. Electroanal. Chem..

[25] Abollino O., Giacomino A., Malandrino M., Piscionieri G., Mentasti E. (2008). Determination of mercury by anodic stripping voltammetry with a gold nanoparticle-modified glassy carbon electrode. Electroanalysis.

[26] Smajdor J., Paczosa–Bator B., Piech R. (2016). Voltammetric Electrode Based on Nafion and Poly(2,3–dihydrothieno–1,4–dioxin)–poly(styrenesulfonate) Film for Fast and High Sensitive Determination of Metamizole. J. Electrochem. Soc..

[27] Dong Y., Ding Y., Zhou Y., Chen J., Wang C. (2014). Differential pulse anodic stripping voltammetric determination of Pb ion at a montmorillonites/polyaniline nanocomposite modified glassy carbon electrode. J. Electroanal. Chem..

[28] Laviron E. (1979). General expression of the linear potential sweep voltammogram in the case of diffusionless electrochemical systems. J. Electroanal. Chem..

[29] Lourencao B.C., Silva T.A., Fatibello-Filho O., Swain G.M. (2014). Voltammetric Studies of Propranolol and Hydrochlorothiazide Oxidation in Standard and Synthetic Biological Fluids Using a Nitrogen-Containing Tetrahedral Amorphous Carbon (ta-C:N) Electrode. Electrochim. Acta.

[30] El-Ries M.A., Abou-Sekkina M.M., Wassel A.A. (2002). Polarographic determination of propranolol in pharmaceutical formulation. J. Pharm. Biomed. Anal..

[31] Pérez Ruiz T., Martínez-Lozano C., Tomás V., Carpena J. (1998). Simultaneous determination of propranolol and pindolol by synchronous spectrofluorimetry. Talanta.

[32] Tabrizi A.B. (2007). A simple spectrofluorimetric method for determination of piroxicam and propranolol in pharmaceutical preparations. J. Food Drug Anal..

[33] Partani P., Modhave Y., Gurule S., Khuroo A., Monif T. (2009). Simultaneous determination of propranolol and 4-hydroxy propranolol in human plasma by solid phase extraction and liquid chromatography/electrospray tandem mass spectrometry. J. Pharm. Biomed. Anal..

[34] Townshend A., Murillo Pulgarín J.A., Alañón Pardo M.T. (2003). Flow injection-chemiluminescence determination of propranolol in pharmaceutical preparations. Anal. Chim. Acta.

[35] Tsogas G.Z., Stergiou D.V., Vlessidis A.G., Evmiridis N.P. (2005). Development of a sensitive flow injection-chemiluminescence detection method for the indirect determination of propranolol. Anal. Chim. Acta.

[36] Dos Santos S.X., Cavalheiro É.T.G., Brett C.M.A. (2010). Analytical potentialities of carbon nanotube/silicone rubber composite electrodes: Determination of propranolol. Electroanalysis.

[37] Shadjou N., Hasanzadeh M., Saghatforoush L., Mehdizadeh R., Jouyban A. (2011). Electrochemical behavior of atenolol, carvedilol and propranolol on copper-oxide nanoparticles. Electrochim. Acta.

